# Production and Purification of Two Bioactive Antimicrobial Peptides Using a Two-Step Approach Involving an Elastin-Like Fusion Tag

**DOI:** 10.3390/ph14100956

**Published:** 2021-09-23

**Authors:** Ana Margarida Pereira, André da Costa, Simoni Campos Dias, Margarida Casal, Raul Machado

**Affiliations:** 1 CBMA (Centre of Molecular and Environmental Biology), Department of Biology, Campus de Gualtar, University of Minho, 4710-057 Braga, Portugal; anamargbp@gmail.com (A.M.P.); andrecosta@bio.uminho.pt (A.d.C.); mcasal@bio.uminho.pt (M.C.); 2 IB-S (Institute of Science and Innovation for Bio-Sustainability), Campus de Gualtar, University of Minho, 4710-057 Braga, Portugal; 3 Genomic Sciences and Biotechnology Program, UCB-Brasilia, SGAN 916, Modulo B, Bloco C, Brasília 70790-160, Brazil; si.camposdias@gmail.com; 4 Animal Biology Department, Campus Darcy Ribeiro, Universidade de Brasília, UnB, Brasília 70910-900, Brazil

**Keywords:** antimicrobial resistance, antimicrobial peptides, recombinant production, elastin-like recombinamer, fusion tag, chemical cleavage

## Abstract

Antimicrobial resistance is an increasing global threat, demanding new therapeutic biomolecules against multidrug-resistant bacteria. Antimicrobial peptides (AMPs) are promising candidates for a new generation of antibiotics, but their potential application is still in its infancy, mostly due to limitations associated with large-scale production. The use of recombinant DNA technology for the production of AMPs fused with polymer tags presents the advantage of high-yield production and cost-efficient purification processes at high recovery rates. Owing to their unique properties, we explored the use of an elastin-like recombinamer (ELR) as a fusion partner for the production and isolation of two different AMPs (ABP-CM4 and Synoeca-MP), with an interspacing formic acid cleavage site. Recombinant AMP-ELR proteins were overproduced in *Escherichia coli* and efficiently purified by temperature cycles. The introduction of a formic acid cleavage site allowed the isolation of AMPs, resorting to a two-step methodology involving temperature cycles and a simple size-exclusion purification step. This simple and easy-to-implement purification method was demonstrated to result in high recovery rates of bioactive AMPs. The minimum inhibitory concentration (MIC) of the free AMPs was determined against seven different bacteria of clinical relevance (*Staphylococcus aureus*, *Staphylococcus epidermidis*, *Escherichia coli*, *Klebsiella pneumoniae*, *Pseudomonas aeruginosa,* and two *Burkholderia cenocepacia* strains), in accordance with the EUCAST/CLSI antimicrobial susceptibility testing standards. All the bacterial strains (except for *Pseudomonas aeruginosa*) were demonstrated to be susceptible to ABP-CM4, including a resistant *Burkholderia cenocepacia* clinical strain. As for Synoeca-MP, although it did not inhibit the growth of *Pseudomonas aeruginosa* or *Klebsiella pneumoniae*, it was demonstrated to be highly active against the remaining bacteria. The present work provides the basis for the development of an efficient and up-scalable biotechnological platform for the production and purification of active AMPs against clinically relevant bacteria.

## 1. Introduction

The emergence of multidrug-resistant pathogens is a worldwide recognized threat. Antimicrobial resistance (AMR) has been driven by the overuse and misuse of antibiotics in humans and animals, having a huge impact on human health and becoming a leading healthcare challenge. Antibiotic-resistant bacteria are alarmingly frequent in hospitals and other healthcare institutions, not only representing a massive economic burden, but also being responsible for millions of deaths worldwide every year [1,2]. The multidrug-resistant Gram-positive bacteria *Staphylococcus aureus* and *Staphylococcus epidermidis*, and the Gram-negative bacteria *Escherichia coli*, *Klebsiella pneumoniae*, *Pseudomonas aeruginosa* [3,4,5,6,7], as well as *Burkholderia cepacia* complex bacteria (Bcc) [8], are some of the most challenging antibiotic-resistant strains, being responsible for various nosocomial infections worldwide, thus prompting the need for new effective therapeutic solutions.

Antimicrobial peptides (AMPs) are promising alternatives to classical antimicrobials. Ubiquitous in nature, these peptides are usually short (<100 amino acids), positively charged (net charge from +2 to +9), and amphipathic molecules, also defined as host defence peptides, due to their role in the innate immunity of many organisms [9,10]. Although the underlying mechanisms of action for AMPs are not entirely clear, the best described mode involves the electrostatic interaction between the cationic peptides and the negatively charged components of the bacterial cell membranes: phosphatidylglycerol (PG) and cardiopolin (CL), phosphate groups in the lipopolysaccharides (LPS) of Gram-negative bacteria, or lipoteichoic acids (LTA) present on the surface of Gram-positive bacteria [9,11,12,13]. This electrostatic interaction leads to the accumulation of AMPs on the surface of the membranes, intercalating with the membrane lipids and resulting in the disruption of membrane integrity [9]. These natural molecules are even more interesting as therapeutic agents, since the action in fundamental structures, such as the bacterial cell membrane, decreases the probability of the emergence of resistance [14,15,16]. Thus, AMPs present several advantages as alternative therapeutic agents to classical antibiotics, and have been the focus of intense research for the past years.

AMPs are generally obtained by extraction from their biological sources or by chemical synthesis, requiring expensive, cumbersome, and low-yield processes, therefore restricting their large-scale production [17,18,19]. The heterologous expression of AMPs using microbial cell factories has the advantage of allowing the production of AMPs in a high-yield, relatively fast and cost-efficient process, with absolute control over the peptide composition [17,18,19]. Indeed, once the initial efforts associated with genetic constructions, and recombinant protein production and purification are surpassed, the process can be easily up-scaled to economically viable levels. Still, the recombinant production of AMPs is challenging, as these peptides are generally toxic for the producing host (e.g., bacteria), can be produced in insoluble inclusion bodies, and are prone to proteolytic degradation, due to their low molecular weight [18]. Fusion tags are often used to overcome these limitations, shielding the peptide from intracellular proteolysis, protecting the host from peptide toxicity, increasing solubility, and facilitating purification [17,18,20,21].

Inspired by mammalian tropoelastin [22], elastin-like recombinamers are characterized by temperature-dependent phase transitional behaviour in water, aggregating and segregating from solution at temperatures above the transition temperature (Tt), in a fully reversible process [22,23]. This particular behaviour represents a promising alternative to traditional chromatographic purification methods, allowing the purification of recombinant proteins by employing simple hot and cold cycles. We have previously demonstrated that an ELR variant, comprising 200 repetitions of the pentamer VPAVG (A200), displays thermal hysteresis, in addition to the phase transition [24]. This protein polymer demonstrated the ability to spontaneously self-assemble into sub-microparticles in aqueous solution, at temperatures above the transition temperature [24,25]. This is highly compelling for purification purposes, as the particle aggregates can be easily collected by centrifugation at high recovery rates, using mild conditions. Moreover, the thermal hysteresis ensures that the self-assembled structures, once formed, are stable over a range of temperatures, avoiding the need for special care in the handling temperature.

In this work, we report the use of A200 as a thermoresponsive protein tag for the production and purification of the following two AMPs, using *Escherichia coli* as a cell factory: ABP-CM4 (CM4), a 35 amino acid antimicrobial peptide, isolated from the haemolymph of the silkworm *Bombyx mori* [26]; and Synoeca-MP (Synoeca), a 14 amino acid antimicrobial peptide, isolated from the venom of the *Synoeca surinama* wasp [27]. CM4 is a member of the cecropin family of peptides, with antibacterial, antifungal, and antitumor activities, while showing no toxicity towards mammalian cells [26,28,29,30,31]. Synoeca is an AMP displaying strong activity against bacteria, including bacterial strains that are resistant to antibiotics, with no significant cytotoxicity against mammalian cell lines [27,32]. The DNA coding sequences of the AMPs were fused to the A200 gene, with an interspacing aspartate–proline (D–P) formic acid cleavage site for the isolation of AMPs from the ELR tag. The antibacterial activity of free, isolated CM4 and Synoeca was assessed against a series of clinically relevant bacteria by determination of the minimum inhibitory concentration (MIC), demonstrating the feasibility of this approach for the production, isolation, and purification of AMPs.

## 2. Results

### 2.1. 3D Structure Analysis of CM4 and Synoeca

**Figure 1:**
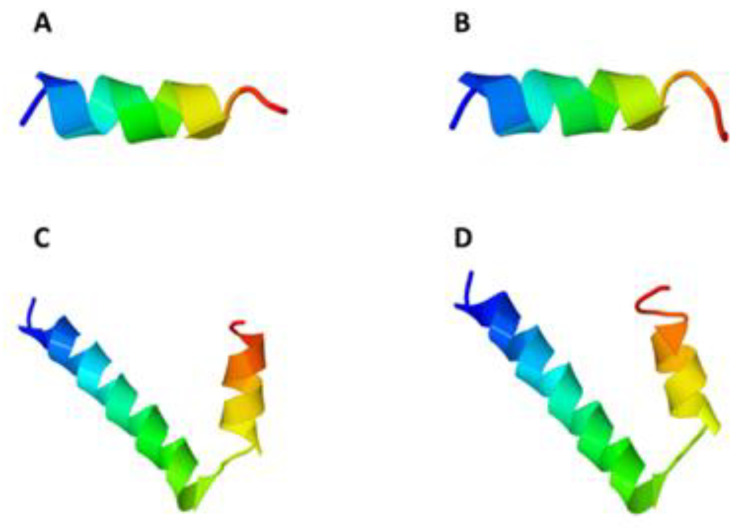
I-TASSER 3D structure prediction of Synoeca without (**A**) and with (**B**) the extra Asp residue, and of CM4 without (**C**) and with (**D**) the extra Asp residue. Images were generated with the Jmol-based Jena3D Viewer (http://jena3d.leibniz-fli.de, accessed on 30 October 2020).

Formic acid specifically cleaves the D–P dipeptide at the C-terminus of the aspartic acid (Asp) residue [33], leaving an additional Asp residue in the recombinant CM4 and Synoeca antimicrobial peptides. Initial studies involved 3D structure analysis to assess if the presence of an extra residue would result in the loss of protein conformation. Three-dimensional structure prediction with the I-TASSER prediction platform showed that the extra amino acid does not modify the overall structure of the AMPs (Figure 1), suggesting that the antimicrobial activity of the peptides is not compromised.

### 2.2. Production and Purification of CM4DP-A200 and SynDP-A200

**Figure 2:**
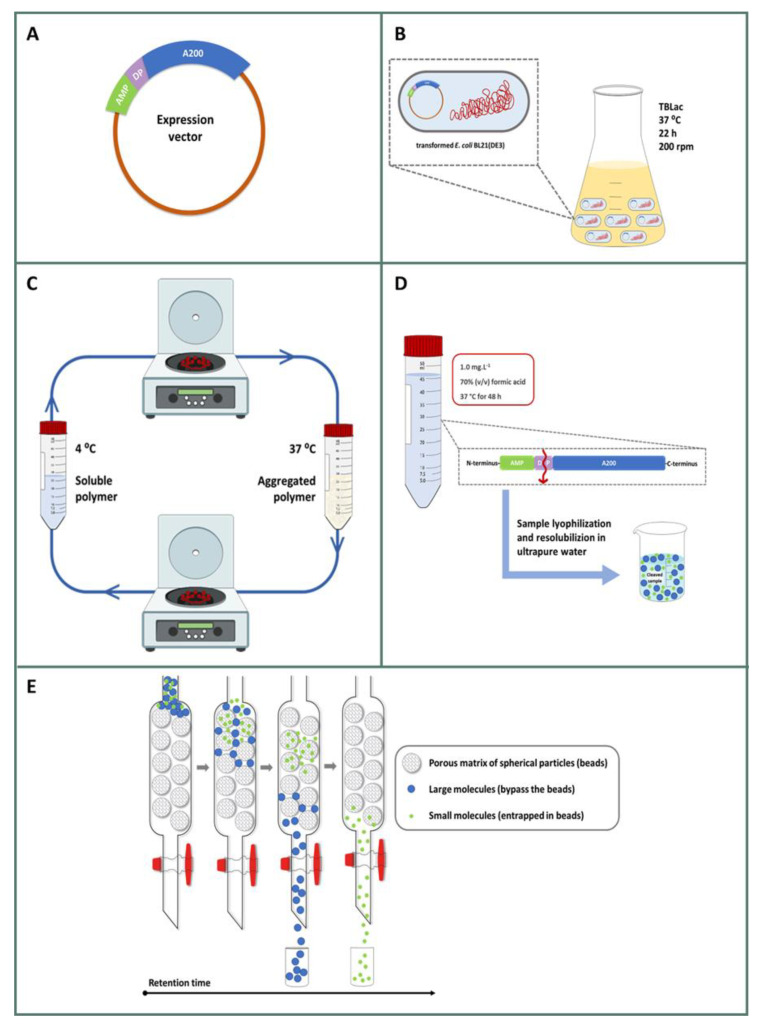
Schematic representation of the overall process used for the production and purification of the antimicrobial peptides. (**A**) AMPs (Synoeca and CM4) are cloned at the N-terminus of A200 previously cloned in an expression plasmid, with the inclusion of a formic acid cleavage site (DP) between the sequences. (**B**) Cells transformed with the expression plasmid are grown in auto-induction medium (TBLac) for 22 h at 37 °C. (**C**) Polymers are purified by inverse transition cycling (ITC) and after lyophilization, (**D**) pure polymers are dissolved in 70% formic acid for protein cleavage for 48 h at 37 °C. Reaction is stopped with mQ water and the sample is freeze-dried and then resolubilized in mQ water. (**E**) AMPs are finally isolated and purified by size-exclusion chromatography (SEC).

The constructions CM4DP-A200 and SynDP-A200 were obtained by the ligation of chemically synthesized CM4 and Synoeca coding sequences, incorporating a formic acid cleavage site (D–P) into an expression vector containing the A200 gene (Figure 2A).

The recombinant proteins CM4DP-A200 and SynDP-A200 were successfully produced in *E. coli* BL21(DE3) by auto-induction (Figure 1B), and purified by employing a pre-acidification step followed by ITC (Figure 1C). The pre-acidification step at pH 3.5 allowed a large number of *E. coli* endogenous proteins to be precipitated, which were further removed by centrifugation, resulting in a clear crude lysate.

**Figure 3:**
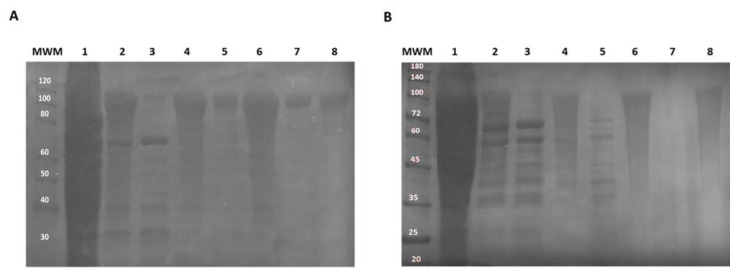
SDS-PAGE of purification by inverse transition cycling for (**A**) CM4DP-A200 and (**B**) SynDP-A200. After sonication of the crude extract (lane 1), lysates were adjusted to pH 3.5 and the precipitates were removed by centrifugation. The supernatants (lane 2) were subjected to three purification cycles of hot and cold incubation and centrifugation steps (lanes 3–8). Due to the thermoresponsive behaviour of A200, both CM4DP-A200 and SynDP-A200 self-assembled and precipitated during incubation in the hot cycle, leaving the contaminants in the supernatant. Lanes 3, 5 and 7 correspond to the supernatants obtained from the first, second and third hot spins, respectively. Lanes, 4, 6 and 8 correspond to the protein-enriched fractions of CM4DP-A200 and SynDP-A200. MWM corresponds to the molecular weight marker in kDa.

Pure recombinant protein fractions were obtained by exploring the thermos responsiveness of the ELR tag using hot and cold incubation, and centrifugation steps (Figure 3). SDS-PAGE analysis of the purification process revealed that some of the CM4DP-A200 was lost during the purification cycles (Figure 3A, lanes 5 and 7). On the contrary, no apparent protein losses were observed for SynDP-A200, indicating very good protein recovery yields. For both proteins, pure protein fractions were obtained after three hot and cold cycles, resulting in volumetric productivities of 77 mg/L and 90 mg/L for CM4DP-A200 (88.8 kDa) and SynDP-A200 (86.8 kDa), respectively. The higher molecular weights observed for the recombinant proteins have been previously observed for ELRs, and are a result of abnormal gel mobility, due to the hydrophobic nature of these proteins [24,25].

### 2.3. Isolation and Purification of Free AMPs

**Figure 4:**
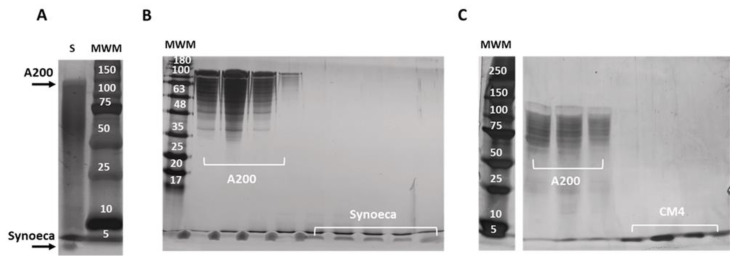
SDS-PAGE of AMP purification by (**A**) inverse transition cycling (ITC) and (**B**) size-exclusion chromatography (SEC) using desalting columns. Purification by ITC (Synoeca used as representative sample) demonstrates the presence of both A200 and AMP in the purified ELR fraction indicating loss of efficiency. Purification by SEC resulted in improved purification efficiency, resulting in high recovery and highly pure (**B**) Synoeca (MW = 1.84 kDa) and (**C**) CM4 (MW = 4.01 kDa) fractions. Gels were stained with silver nitrate. MWM—molecular weight marker (KDa).

For the isolation of the AMPs from the ELR tag, the pure fusion proteins (CM4DP-A200 and SynDP-A200) were subjected to chemical cleavage by formic acid (Figure 2D), and subsequently isolated from the ELR tag by ITC—that is, by employing hot and cold cycles. The SDS-PAGE analysis of purification by ITC demonstrated that the process was not completely efficient, as the precipitated ELR fraction still contained a significant amount of AMP (Figure 4A). For this reason, we devised an improved protocol for the purification of AMPs, based on the principle of size-exclusion chromatography. After chemical cleavage with formic acid, the lyophilized mixture of AMP and A200 was resuspended in cold mQ water and loaded into a desalting column (Figure 2E). This method has demonstrated to be efficient, resulting in highly pure peptide fractions for both Synoeca (Figure 4B, MW = 1.84 kDa) and CM4 (Figure 4C, MW = 4.01 kDa). The data obtained by MALDI-TOF confirmed the molecular weight of the purified AMPs, which is consistent with the theoretical values (Table 1). After purification and lyophilization, the optimized process resulted in final yields of 1.5 mg and 0.5 mg of free CM4 and Synoeca per 100 mg of fusion protein, respectively.

### 2.4. Antibacterial Activity of Free AMPs

**Figure 5:**
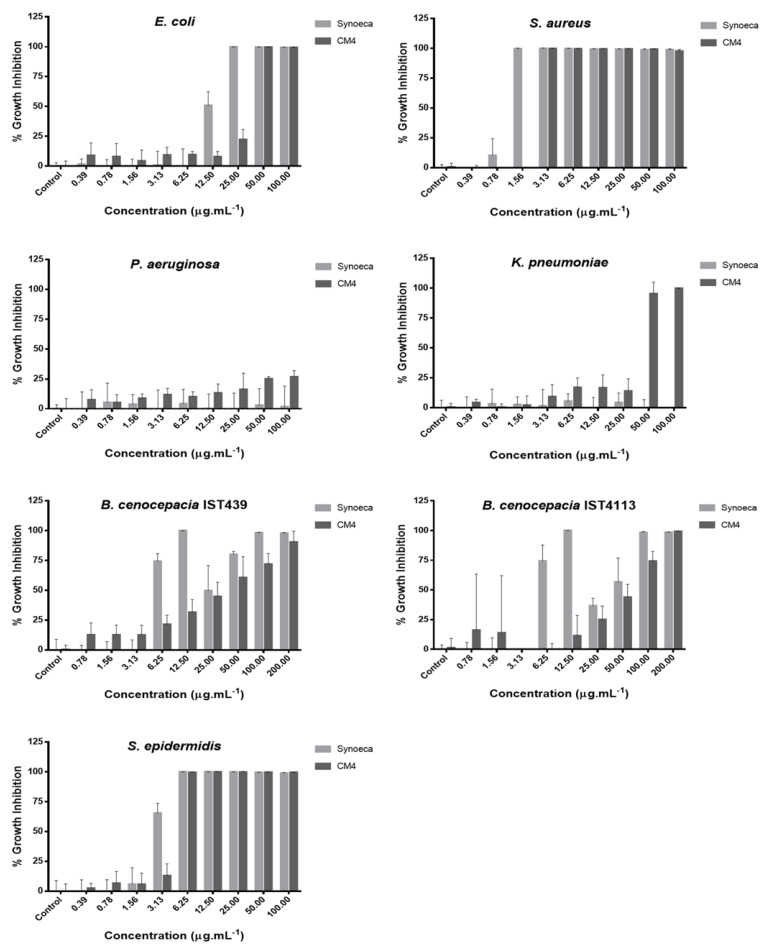
Antibacterial activity of Synoeca (light grey) and CM4 (dark grey) against different bacteria after 18 h of incubation at 37 °C. Bars indicate the mean of at least three replicates ± standard deviation (SD).

The antibacterial activity of free CM4 and Synoeca was evaluated by determination of the minimum inhibitory concentration (MIC) against Gram-negative (*E. coli* HB101, *P. aeruginosa* ATCC10145, *B. cenocepacia* IST439, *B. cenocepacia* IST4113, and *K. pneumoniae*) and Gram-positive (*S. aureus* ATCC6538 and *S. epidermidis*) bacteria. The AMPs were incubated with bacterial suspensions and assessed for bacterial growth, with the MIC defined as the lowest concentration of AMPs that inhibited the visible growth of bacteria (Figure 5).

Table 2 summarizes the MIC values obtained for Synoeca and CM4 against the testing bacteria. Within the range of concentrations tested, the results show that all the bacterial strains, except for *P. aeruginosa,* are susceptible to CM4, with the lowest MIC values being found for *S. aureus* (3.13 mg/L), S. epidermidis (12.50 mg/L), *E. coli* (50.00 mg/L), and *K. pneumoniae* (50.00 mg/L). While *P. aeruginosa* demonstrated a mean value of growth inhibition as low as 25% for a concentration of 100.00 mg/L, the B. cenocepacia strains show a clear trend of increased growth inhibition with increasing concentration, reaching values of around 75% for a concentration of 100.00 mg/L. Therefore, for these specific strains, the range of testing concentrations was further extended to 200.00 mg/L, reaching mean values of growth inhibition of 90% and 100% for *B. cenocepacia* IST439 and *B. cenocepacia* IST4113, respectively. As for Synoeca, the lowest MIC values were found for *S. aureus* (1.56 mg/L), *S. epidermidis* (6.25 mg/L), *B. cenocepacia* IST439 (12.50 mg/L), *B. cenocepacia* IST4113 (12.50 mg/L), and *E. coli* (25.00 mg/L); whereas, no MICs were found for *K. pneumoniae* and *P. aeruginosa,* with mean values of growth inhibition lower than 5%. Comparing the MIC results, the values obtained with Synoeca for *E. coli*, *S. aureus,* and *S. epidermidis* are half of those obtained with CM4, and sixteen times lower against the two *B. cenocepacia* strains.

## 3. Discussion

In this study, we explored the use of the elastin-like recombinamer A200 as a purification tag for the production and isolation of Synoeca and CM4. These AMPs display strong antimicrobial activity against a wide range of bacteria, with no significant cytotoxicity against mammalian cells [26,27,29,30,31,32], thus representing the opportunity to develop new antibiotic treatments to tackle antimicrobial resistance. The AMPs were fused to the N-terminus of the ELR A200, with an interspacing formic acid cleavage site, to enable the isolation and purification of the AMPs. The recombinant protein polymers have demonstrated to be overexpressed in auto-induction media and, owing to the thermoresponsive properties of A200, protein purification was achieved through hot and cold incubation, and centrifugation cycles. We have previously demonstrated that A200 displays reversible thermal transition behaviour, self-assembling into slightly ellipsoidal submicroparticles at temperatures ~33 °C [24,34], and resolubilizing at low temperatures (i.e., below 10 °C). In this work, the ITC process has demonstrated to result in pure polymer fractions, although with some recovery losses during the heating cycles. This is likely due to the ability of AMPs to aggregate and self-assemble into supramolecular nanostructures, resulting from their amphipathic nature [35]. From our experience, recovery yields can be improved by the addition of low amounts of salt (results not shown); however, this can also result in the loss of antimicrobial activity of the peptides [36,37,38]. Nevertheless, this simple and facile approach has great advantages over other expensive, cumbersome, and time-consuming techniques, such as immobilized metal affinity chromatography (IMAC).

After purification by ITC, the fusion proteins were subjected to chemical cleavage with formic acid, to isolate the AMPs from the ELR tag. Chemical cleavage presents advantages over enzymatic cleavage, such as cost effectiveness and suitability for industrial production. Also, due to the small size, the chemical agents are able to access the cleavage sites of fusion proteins that are sometimes inaccessible to bulky proteases. Here, formic acid was chosen for the selective cleavage at the aspartic acid–proline (D–P) peptide bond, due to the efficiency and specificity of the process [39]. After formic acid cleavage, the mixture of cleaved proteins (AMP and ELR) was subjected to ITC, to separate the peptide from the ELR tag. During inverse transition cycling, the ELR polypeptide chains self-assemble at temperatures above its Tt, acquiring a more ordered structure and segregating from the solution [24,25]. This allows the ELR to be collected by centrifugation, leaving the free AMP in the supernatant. Nevertheless, the formation of the self-assembled structures in the hot cycle can result in the entrapment of molecules within the protein polymer matrix [34,40,41], thus reducing the overall efficiency of AMP recovery and explaining the presence of cleaved AMPs in the ELR-enriched fraction (Figure 4A). To improve the recovery efficiency of the purification process, we resorted to a simple size-exclusion chromatography process, using no other buffer, but water as mobile phase. After being loaded into a column packed with a resin consisting of a porous matrix of spherical particles (beads), the biomolecules were separated based on their sizes. Thus, small molecules, such as AMPs, became temporarily trapped within the pores of the beads, while larger molecules, such as ELRs, with high molecular weights, pass around the beads and are eluted first (Figure 2E). This additional simple downstream processing has demonstrated to greatly improve the overall recovery of the AMPs (Figure 4B,C), resulting in yields of ca. 1.8-times higher than those previously reported for the biosynthesis of CM4 using an ELP based on the VPGVG sequence as a fusion protein and an intein-mediated self-cleaving tag [42]. To the best of our knowledge, this is the first report on the recombinant production of an active Synoeca antimicrobial peptide.

The antimicrobial activity of the free AMPs was assessed via MIC determination, according to EUCAST [43] and CLSI [44] antimicrobial susceptibility testing standards, against bacteria of high clinical relevance, namely, *Escherichia coli*, *S. aureus*, *P. aeruginosa*, *K. pneumoniae*, *S. epidermidis,* and two clonal variants of *B. cenocepacia*. Analysis of the MIC results reveals significant differences between the potency of the peptides. Although both AMPs were effective in completely inhibiting the growth (100% growth inhibition) of *S. aureus*, *S. epidermidis,* and *E. coli*, Synoeca has demonstrated to be more potent than CM4, showing MIC values that were two-fold lower. In contrast, CM4 has demonstrated to inhibit the growth of *K. pneumoniae,* whereas Synoeca lacked antimicrobial activity. As for *P. aeruginosa*, both AMPs demonstrated no or low potency. One possible explanation could be attributed to biofilm formation during the assay, hampering the access of AMPs to the bacterial cells. *P. aeruginosa* bacteria are well known for being able to form robust biofilms, making them less susceptible to antimicrobial agents [45,46]. In such a situation, the positively charged AMPs might have interacted preferentially with negatively charged components of the *P. aeruginosa* biofilm matrix (proteins and extracellular polymeric substances [45,46]), hindering the contact of AMPs with the bacterial membrane [47].

A remarkable result is the activity of both Synoeca and CM4 against the *B. cenocepacia* isolates, completely inhibiting the growth of the tested bacteria. Isolate IST439 was obtained from a CF patient, while the clonal variant IST4113 was collected 3 years later from the same patient, after a period of exacerbated infection and intravenous antibiotic therapy. The latter has demonstrated to be more resistant, as a consequence of the up-regulation of numerous genes/proteins that are crucial for the genetic adaptation of bacteria, to overcome the inhibitory effects of antimicrobial agents [8,48,49,50]. Nevertheless, the AMPs were able to inhibit the complete growth of *B. cenocepacia*.

## 4. Materials and Methods

### 4.1. Biological Materials

*E. coli* XL1-Blue and BL21(DE3) strains were used for the cloning and production steps, respectively. *E. coli* HB101, *S. aureus* ATCC6538, *P. aeruginosa* ATCC10145, *B. cenocepacia* IST439, *B. cenocepacia* IST4113, and clinical isolates of *K. pneumoniae* and *S. epidermidis* were used in the broth microdilution MIC assays. The clonal variants of *B. cenocepacia* were isolated, with a 3-year difference, from the same cystic fibrosis (CF) patient [51].

### 4.2. 3D Structure Analysis

The 3D structure of the AMPs was predicted using the iterative threading assembly refinement (I-TASSER) server platform [52]. Peptide models were obtained from I-TASSER after submission of the amino acid sequences (http://zhanglab.ccmb.med.umich.edu/I-TASSER, accessed 30 October 2020). Images were generated after uploading I-TASSER files onto Jmol-based Jena3D Viewer website (http://jena3d.leibniz-fli.de, accessed 30 October 2020) [53].

### 4.3. Gene Construction and Protein Production

The genetic constructions for CM4DP-A200 (MW = 89.0 kDa) and SynDP-A200 (MW = 86.8 kDa) were obtained following previously described methodologies [30]. Briefly, AMPs containing the aspartate–proline (D–P) formic acid cleavage sequence were chemically synthesized with flanking *Nde*I and *Kpn*I restriction sites (GenScript) and cloned at the N-terminus of the ELR A200 polymer previously cloned in a modified pET25b(+) (Novagen) expression plasmid [24]. The final expression plasmids (Figure 2A) were confirmed by DNA sequencing (Eurofins) and transformed into *E. coli* BL21(DE3) for recombinant protein production (Figure 2B). The cells harbouring the expression plasmids were grown in shake flasks for 22 h at 37 °C and 200 rpm, using Terrific Broth (12 g tryptone, 24 g yeast extract, 5.04 g glycerol, 12.54 g K_2_HPO_4_, 2.31 g KH_2_PO_4_, per litre), supplemented with 50 mg/L kanamycin and 2 g/L lactose to allow for auto-induction (TBlac), and a culture volume to flask volume ratio of 1:4 [54]. After protein production, bacterial cells were collected by centrifugation, resuspended in TE buffer solution (50 mM Tris-HCl + 1 mM EDTA at pH 8.0) and disrupted by sonication using a Vibra cell™ 75043 (Bioblock Scientific) with a 25 mm diameter probe (3 s pulse on, 9 s pulse off, total sonication time: 10 min). Samples were kept at ice-cold temperature during the entire process. The pH of the crude lysate was then adjusted to pH 3.5 with 1.6 M HCl to precipitate endogenous *E. coli* contaminants [25], followed by centrifugation at 11.500× *g*, for 20 min at 4 °C. The clear supernatant was purified by inverse transition cycling (ITC) consisting of 3 cycles of hot (37 °C) and cold (4 °C) incubation (2 h at each temperature) and centrifugation steps (11,500× *g*, 20 min) (Figure 2C). The purification steps were monitored by 10% sodium dodecyl sulphate-polyacrylamide gel electrophoresis (SDS-PAGE) with 0.3 M copper chloride staining. The purified polymer fraction was lyophilized (Christ Alpha 2-4 LD Plus from Bioblock Scientific, Illkirch Cedex, France) and stored at −20 °C before use.

### 4.4. Cleavage and AMP Isolation

The CM4 and Synoeca peptides were isolated from the ELR tag by chemical cleavage with formic acid [30,33]. The protein polymer was resuspended in formic acid 70% (*v*/*v*) with a final concentration of 1.0 mg/mL, and incubated for 48 h at 37 °C. The reaction was stopped by addition of three times the volume of the initial reaction with ultrapure water (mQ; Milli-Q^®^, Millipore, Burlington, MA, USA). The samples were then frozen and lyophilized. The cleaved AMP was purified from the mixture containing the peptide and the ELR tag by size-exclusion chromatography, using a HiPrep 26/10 desalting column (GE Healthcare). For this, the lyophilized samples containing the cleaved fractions were resuspended in cold mQ water in a concentration of 5 mg/mL and loaded into a pre-equilibrated column. The elution buffer (cold mQ water) was applied to the column and fractions were collected and analysed by SDS-PAGE (10% gel), followed by silver staining. The overall process is schematically represented in Figure 2D,E. Finally, the fractions containing pure AMPs were frozen, lyophilized, and stored at −20 °C.

### 4.5. MALDI-TOF Mass Spectrometry

The mass/charge of pure isolated (cleaved) CM4 and Synoeca peptides was verified by matrix-assisted laser desorption/ionization with time of flight (MALDI-TOF) on an Ultra-Flex MALDI-TOF instrument (Bruker Daltonics GmbH, Bremen, Germany) equipped with a 337 nm nitrogen laser and using sinapic acid as the matrix (≥99.5%). Sample preparation was performed as previously described [55].

### 4.6. Minimum Inhibitory Concentration (MIC)

The antibacterial activity of the isolated AMPs was assessed via broth microdilution method to determine the MIC against clinically relevant Gram-negative and Gram-positive bacteria—*E. coli* HB101, *P. aeruginosa* ATCC10145, *B. cenocepacia* IST439, *B. cenocepacia* IST4113, *K. pneumoniae* isolate, *S. aureus* ATCC6538 and *S. epidermidis* isolate—following the European Committee on Antimicrobial Susceptibility Testing (EUCAST) and the Clinical Laboratory Standards Institute (CLSI) guidelines [43,44]. A detailed description of the method is provided by Wiegand et al. [56]. Briefly, stock solutions of 2 mg/mL of AMP in sterile mQ water were used for the preparation of two-fold serial dilutions in MHB, to obtain final testing concentrations within the range of 0.39–100 mg/L (200 mg/L for *B. cenocepacia* strains). For the MIC assays, 50 μL of protein solution and 50 μL of bacterial suspensions in MHB (1 × 10^6^ CFUs/mL) were mixed in 96-well plates (Nunc™ Edge 2.0 96-well plates, Thermo Scientific™, Waltham, Massachusetts, EUA) and incubated for 18 h at 37 °C. Growth inhibition was assessed by measuring optical density at 600 nm (OD_600nm_). The MIC was determined as the lowest concentration of antimicrobial peptide that inhibited the visible growth of the testing bacteria. At least three independent replicates were performed for each peptide and bacterial strain. Data were analysed by GraphPad Prism 6 Software (GraphPad Software, Inc., La Jolla, CA, USA).

## 5. Conclusions

We have developed an efficient and cost-effective approach for the recombinant production and purification of bioactive AMPs, with high protein recovery. The recombinant fusion proteins CM4DP-A200 and SynoecaDP-A200 were efficiently produced in *E. coli,* and purified from the cell crude lysate by a simple non-chromatographic process involving temperature cycles. This allows not only the purification of the recombinant fusion proteins, but also the concentration of these so as to minimize volume handling. The adoption of a size-exclusion purification step, using desalting columns, was demonstrated to be an efficient, up-scalable and simple process to isolate the free AMPs from the ELR tag, with high recovery. The purified AMPs—Synoeca and CM4—demonstrated antimicrobial activity towards clinically relevant Gram-positive and Gram-negative bacteria, making them promising antibacterial agents. This study contributes support to push forward the recombinant production of AMPs and their potential clinical application.

## Figures and Tables

**Table 1:** Theoretical (Expasy ProtParam; https://web.expasy.org/protparam/, accessed on 30 October 2020) and experimentally determined (MALDI-TOF) molecular weights (in Da) for pure isolated Synoeca and CM4.

Antimicrobial Peptide	Theoretical	*m*/*z* (MALDI-TOF)
Synoeca	1843.30	1872.39
CM4	4008.78	4020.90

**Table 2:** Synoeca and CM4 MICs against different bacteria.

Bacterial Species	MIC (mg/L)	
	Synoeca	CM4
*Escherichia coli*	25.00	50.00
*Staphylococcus aureus*	1.56	3.13
*Klebsiella pneumoniae*	>100.00	50.00
*Pseudomonas aeruginosa*	>100.00	>100.00
*Burkholderia cenocepacia* IST439	12.50	~200.00
*Burkholderia cenocepacia* IST4113	12.50	200.00
*Staphylococcus epidermidis*	6.25	12.50

## Data Availability

The data presented in this study are available in the article.
